# Supplementary material for: Inductive Game Theory and the Dynamics of Animal Conflict
Source: PLoS Comput Biol. 2010 May 13;6(5):e1000782. doi: 10.1371/journal.pcbi.1000782 (PMC2869306; doi:10.1371/journal.pcbi.1000782)
Supplement: Text S1 — Supporting information for the main MS. (0.16 MB PDF) [file pcbi.1000782.s001.pdf]

Supporting Information for Inductive Game Theory and the Dynamics of Animal Conflict

Simon DeDeo[1,2], David C. Krakauer[1], Jessica Flack[1,3,*]
1 Santa Fe Institute, Santa Fe, NM 87501, USA
2 Institute for the Physics and Mathematics of the Universe, University of Tokyo, Kashiwa-shi, Chiba 277-8582, Japan
3 Santa Fe Institute, Santa Fe, NM 87501, USA & Yerkes National Primate Research Center, Atlanta, GA 30322, USA
*** E-mail: jflack@santafe.edu**

Aggregate Properties of the Time Series

To explore how memory might influence collective conflict dynamics, we determine the scales of aggregation in our study group at which significant correlations in conflict properties occur. We test for spurious correlations, which can arise without memory if the same individuals repeatedly engage in conflict simply because they have an aggressive disposition or if particular individuals repeatedly agitate others.

We first consider the simple case of correlations across fights in the number of individuals involved without regard to identity – for example, whether large fights follow large fights. We find that, for a wide range of such statistics involving both the median and mean, and different nulls, that there are no detectable correlations of this sort. This section elaborates on that finding.

Consider the statistic "median over-under correlation," an average taken over all fight pairs separated by time Δt, which can be defined and computed with ensemble averages:

$$M(\Delta t) = \langle \Theta(N[t] - \bar{N})\Theta(N[t + \Delta t] - \bar{N})\rangle, \tag{1}$$

where the angle-bracket average is taken over the entire dataset but no pairs with fights on different days are considered. Here Θ is a step-function: -1 for negative argument and +1 for positive argument. In the case where the fight size of one of the pairs happens to be precisely the median value, *i.e.*, the argument is zero, we take the Θ function to be $(n-m)/(n+m)$, where n is the number of fights with length strictly above the median value, and m the number of fights strictly below the median value. The time, t, and lag, Δt, are in units of fights within a day; for example, t of five means the fifth fight that day.

The average fight size, $\bar{N}$, can computed within a day or across days. We find evidence for intra-day variation associated with feeding times and onset of evening hours; however, using a time-dependent $\bar{N}$ complicates analysis without changing any qualitative aspects of the results, and so we do not use this correction. Put simply, then $M(\Delta t)$ estimates how likely a fight of above-median (equivalently, below-median) size is expected to be found after a previous fight also of above-median (below-median) size.

Any correlation statistic such as M will, in general, be non-zero for a particular data set – even if the data were generated by a completely uncorrelated process. In order to determine the significance level of a detection, we must compare to a null model. Here our null model is a shuffled time-series that leaves the internal properties of the fights unchanged. This null has the advantage of efficiently ensuring that detections will be sensitive to purely time-correlated aspects of the data, and not – for example – to the "zero-lag" correlations that occur within a single fight or on average. In general, we find our statistics insensitive to whether this shuffling is done within a day – *i.e.*, keeping same day events on the same day but rearranging their order – or across all days.

As shown in Fig. 1, there is no evidence for memory of previous interactions when considering only fight size data. Conflicts at this coarse-graining appear to occur at random. Furthermore, the correlations of fight sizes, fight durations, and peace durations with themselves and with each other (variations of $M(\Delta t)$ sensitive to, for example, whether a long peace follows a small fight) all appear largely independent

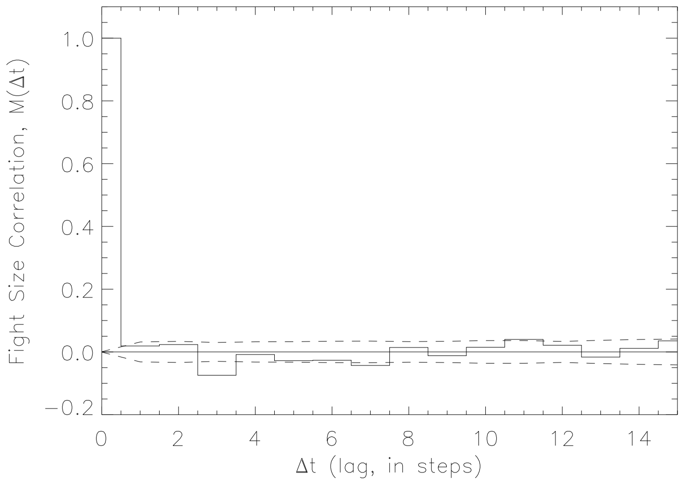

Figure 1. Fight size, an aggregate-level variable, does not predict future fight size. A coarse-grained correlation function, $M(\Delta t)$, of the time series. At lag of zero, M is unity by definition; at non-zero lag, M is indistinguishable from the null model with zero time correlation – the 68% confidence range for this null is shown as the dashed line. The null model is generated by time shuffling fight bouts.

of previous events. This result is robust regardless of whether we examine the correlation function in real space or, conversely, in Fourier space where longer baseline trends, such as those involving a small random walk component, might have become apparent.

The distribution of fight sizes is a central part of our study; as shown in the "Conflict Cost" subsection of the Results, it has important consequences for individuals as well as the group as a whole. Larger fights lead to greater amounts of injury and aggression; meanwhile, the consequences of different strategies for fight size distributions are shown in Figs. 4 and 7 of the main paper. Here, we plot the distribution of all fight fight sizes (on a linear scale) in Fig. 2 (overleaf); in Fig. 3 we plot the distribution for the subset of those fights that include the most common fight participant (individual name Eocene, codename "Eo.")

Individuals and Subgroups in the Time Series

At a finer level of resolution we consider correlations across fights in membership considering all 48 socially-mature individuals. This co-occurrence time-series describes which individuals, pairs, and higher-n groups of individual appeared together in fights, regardless of their behavior.

Because our choice of null conserves the properties of individual fights, $N(A)$ is the same for both the data and the nulls. Given a sufficiently large set of Monte Carlo realizations of the null, we can determine which pairs of individuals have significant time correlations – *i.e.*, have a ΔP sufficiently positive or negative that the ΔP is shared by less than, *e.g.*, 5% of all Monte Carlo realizations of the null model.

Considering only the group's 48 socially-mature individuals, there are 2,304 possible correlations of the form $\Delta P(A \to B)$. Results are shown in the (implicitly) causal network in Fig. 2. Many of the strongest correlations are positive, meaning that individuals attract one another to subsequent conflicts, rather than negative, which would mean individuals are inhibited from appearing after certain other individuals appeared.

It is striking the extent to which the absence of signal at the most coarse-grained level of the conflict

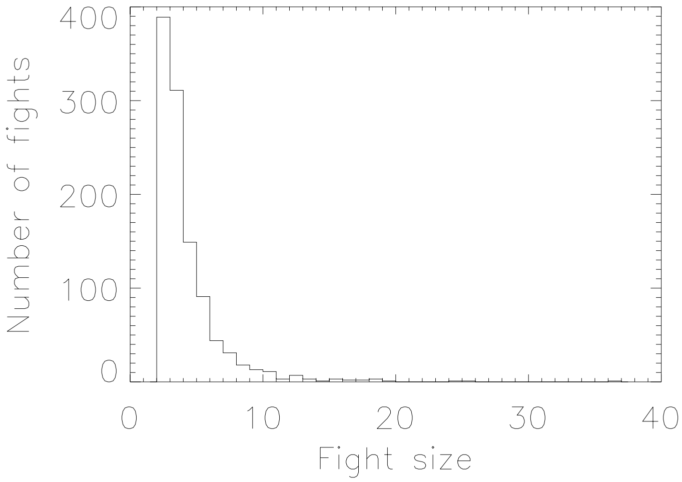

Figure 2. Distribution of fight sizes in the data set. Maximum fight size is 36, mean is 3.7, median is 3.

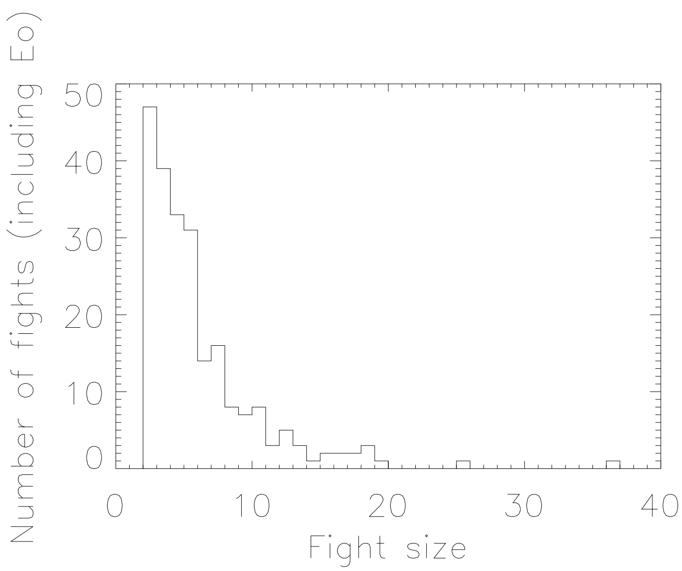

Figure 3. Distribution of fight sizes in the data set, for those fights including the most common individual, code name "Eo" (Eocene.) maximum is 36, mean is 5.6, median is 4.

time-series, Fig. 1, conceals a rich set of correlations once individual identities are considered, Fig. 2. As mentioned, however, extracting overall significance levels for ΔP measurements requires caution. For example, since individuals are correlated *within* fights, and these correlations are maintained by the null model, the various ΔP measurements are not independent of each other.

The effects of these correlations can be seen when comparing the analysis done on the data to that performed on a shuffled version of the data. Taking only those cases where $N(B|A)$ is larger (or smaller) than 95% of $N_{\rm null}(B|A)$ found, there are 513 detections of non-zero ΔP in the data. There are however

approximately 480 "detections" out of 2304 $\Delta P(A \to B)$ for the shuffled data – if the different $N(B|A)$ were perfectly independent, this should be a factor of two lower.

Detections of the positive ΔP alone are more reliable; we find 296 ΔP values larger than zero in the data, compared to an average of 212 in shuffled datasets, for an overall $p < 0.001$. When we come to consider the two-step correlations, the ΔP can not be distinguished from the noise we expect from the internal correlations of the null model. We defer more detailed discussion of these issues to later work.

The distributions of $\Delta P(A \to B)$, $\Delta P(AB \to C)$, and $\Delta P(A \to BC)$ are shown in Fig. 4; these statistics will play a central role in modeling the group, and in assessing how well the model reproduces the group behavior. In Fig. 5 we show the distribution of the "absolute" probabilities P from which the ΔP are derived ($P(A \to B)$ is equal to $N(B|A)/N(A)$, and so forth.)

The null here, recall, is the average from a large Monte Carlo set of null models generated by time-shuffling the series but leaving fight compositions intact. It corresponds to a Markov process where the group has a particular set of conflicts of different compositions that it visits in a biased but uncorrelated fashion. It can be thought of as drawing a fully specified fight from an urn without replacement.

It is possible to consider relaxing the null model further, so that, for example, individuals join fights with a probability independent of the other members of the group; in this case, one assembles a fight by drawing, with probabilities $P(A)$, N individuals, where N might be, for example, drawn from the observed distribution of fight sizes. Such a model destroys many of the correlations, internal to a fight, that are detectable in the time-averaged properties of the data.

This can be seen in Fig. 6, which compares the observed joint probability, $P(AB)$, and the probability expected if individuals appeared independently of each other, $P(A)P(B)$. A two-sided Kolmogorov-Smirnov test finds the two distributions different with $p \ll 10^{-6}$. Such strong rejections of nulls that ignore correlations between individuals are a general feature of our system, which is strongly correlated both within and between fights.

Simulation Specification

For a strategy class $\mathcal{C}(n, m)$, we specify two things. First the probabilities, associated with a particular n- and m-tuple [1], that the n-tuple recommend the m-tuple "join," or "avoid" the subsequent fight. In our study here, we do not develop algorithms to search and "fit out" the many thousand-dimensional space, but make a good first guess to associate the measurable ΔP from the simulations with these probabilities. A positive ΔP for a particular n and m-tuple pair is read as a probability to generate a recommendation to join. A negative ΔP is read as the negative of the probability of a recommendation to avoid.

A ΔP of zero (*i.e.*, a ΔP that could not be detected above a particular confidence level) never generates a recommendation; in many cases, the confidence level requirement zeros out ΔPs that may be formally large – this is because we exclude measurements of a particular ΔP that rely on two or fewer instances of fight pairs.

We have checked and find our results insensitive to reasonable changes in the ΔP cutoff – either in amplitude or in the number of observations required to establish it. The dynamics of conflict are largely driven by the tuples with easily measured, larger ΔP.

For each possible m-tuple in the population, we "roll the dice" for all the n-tuples in the previous fight. For example, for a fight with four participants, there are four 1-tuples, six 2-tuples, four 3-tuples, and one 4-tuple. If we are considering a strategy of $\mathcal{C}(2, 1)$, we are concerned with the six 2-tuples, and we will have, for each of them, either a recommendation to join, a recommendation to avoid, or no recommendation at all, for each of the 48 possible outgoing 1-tuples.

The next step is to combine these. We consider two simple choices of combinator. The recommendations can combined with an `AND` function, requiring a 'recommendation to join' from all relevant n-tuples

[1] Note that we use "tuple" here informally, to mean an unordered set of a particular size.

at the previous step; here 'relevant' means "with a non-zero ΔP". Or, the recommendations can be combined with an `OR` function, *i.e.*, needing at least one n-tuple in the previous time step to recommend 'yes.'

An interpretation of the `AND` rule is conservatism – that individuals who require many recommendations, or recommendations from all previous conflict participants, to join a subsequent fight are conflict averse. In contrast, an interpretation of `OR` is that individuals who require one or only a few recommendations are pushed over the edge more easily – they are conflict prone.

Given both a model and a combinator, we can now simulate the group's conflict dynamics. We start with a seed pair drawn from the distribution of pairs in the data (we take the distribution of "fights with only two individuals", rather than $P(AB)$, but find our results largely insensitive to this choice.) We evaluate the strategies for each individual in the group to determine the composition of the next fight. At some point, a particular fight will, through the propagation effects generated by playing particular strategies, lead to no recommendations for subsequent m-tuples to join. At that point, the "cascade" is terminated, and a new seed is chosen. As shown in Table 1, we consider six different possibilities within the simplest subset of cognitively plausible models.

Our model is not exhaustive, and there are a number of "limit cases." Many of them focus on how to handle joint "avoid" and "join" recommendations for the same m-tuple. In the `OR` combinator case, for instance, does the appearance of a single "avoid" recommendation force the tuple to avoid the next fight, or is it overruled by a single "join" recommendation? We find that the role of the negative ΔPs is very minor; in particular, it is rare that a recommendation to avoid frustrates the appearance of a tuple that received recommendations to join from other tuples. This seems to indicate that non-appearance in a fight is associated more with disinterest, rather than active decisions based on fear or calculation that lead to successful avoidance.

Another limit case is the situation in which an individual receives a recommendation to join, but no other individual does: the individual "can find nobody who wants to fight." This might depict a behavioral reality, or, conversely, it might indicate the need for new dynamical rules; the single individual may be able to generate conflict in ways not detectable by our segmentation of the data into "fight" and "peace" bouts. This question is necessarily limited by our coarse-graining of the continuous and complex sequence of events – individual expressions of aggression over a spatially extended area.

Composition Statistics

We use a set of statistics to quantify how well different variants of $\mathcal{C}(2,1)$ reproduce the data. We consider $P(A)$, the frequency of appearance of individual A, and $P_c(AB)$, the connected pair correlation,

$$P_c(AB) = P(AB) - P(A)P(B), \tag{2}$$

where $P(AB)$ is the frequency of appearance of the pair $P(AB)$. (The use of only the connected part reduces the covariance of the two statistics.)

We also consider $\bar{n}(A)$ and $\bar{n}(AB)$, the size of the average fight in which one finds individual A, or pair AB, respectively:

$$\bar{n}(A) = \frac{\sum_{i=2}^{48} iN(A,i)}{N(A)\bar{n}} \tag{3}$$

and

$$\bar{n}(AB) = \frac{\sum_{i=2}^{48} iN(AB,i)}{N(AB)\bar{n}} \tag{4}$$

where $\bar{n}$ is the average fight size overall, $N(A,i)$ is the number of fights that include A of size i, and similarly for $N(AB,i)$. Note that we normalize $\bar{n}(A)$ and $\bar{n}(AB)$ to the average fight size so that, for

Table 1. The implications for social stability of different strategies.

Strategy	Hypothesis	Combinator	Avg. Max. Size	Avg. Length	Consequences
$\mathcal{C}$(1,1)	"Rogue Actor"	+`OR`	2.03	1.15	Anomalous Quiescence
		+`AND`	2.02	1.10	Anomalous Quiescence
$\mathcal{C}$(1,2)	"Triadic Coordination"	+`OR`	~ 40 *	$\gg 10^2$ *	Forest Fire
		+`AND`	19.9	108	Forest Fire
$\mathcal{C}$(2,1)	"Triadic Discrimination"	+`OR`	5.59	3.73	Forest Fire
		+`AND`	2.89	2.12	Manageable

The severity of conflict dynamics, either in terms of average maximum fight size, or average cascade length, varies strongly depending on the model. Column six gives the consequences for the group of a particular model: anomalous quiescence (few large fights), forest fires (long cascades and large fights), or "manageable" (no extremely large fights). Memory is pair-wise for $\mathcal{C}(1,1)$ and is triadic for $\mathcal{C}(1,2)$ and $\mathcal{C}(2,1)$. $\mathcal{C}(1,2)$ requires that two individuals jointly engage in conflict at the next time step. $\mathcal{C}(1,1)$ and $\mathcal{C}(2,1)$ do not require joint action. $\mathcal{C}(2,1)$ requires that individuals discriminate pairs rather than just individuals. As n and m increase, cognitive burden increases because individuals must remember more conflict participants. An asterisk indicates that fights grew so large that reliable statistics were computationally infeasible.

example, $\bar{n}(Q)$ equal to 2 means that the average fight individual Q appears in is twice the size of an ordinary fight.

Finally, we consider the long fraction, introduced in Eq. 4 of the main paper, and the causal statistic $\Delta P(A \to B)$.

Likelihood Estimation for Model Comparison

In the main paper we used the Pearson correlation as a way to determine how well different strategies reproduced the group's behavior. That the base model (and coarse-grained versions with $n \geq 2$) generally outperformed the other model variants we took as evidence for the importance of the triadic nature of the strategies.

Here, we take a further step towards quantifying the ways in which our model, and competing variants, reproduce the data. We use the (natural) logarithm of the likelihood ratio per measured parameter, $\Delta\mathcal{L}/n$. This gives an estimate of the relative likelihood of the data being produced by one model versus another. If $\Delta\mathcal{L}/n$ is α, then, for an average measurement, the base model is $e^{-\alpha n}$ times more likely than the variant in question.

The calculation of $\Delta\mathcal{L}$ requires an estimate of the shape of the likelihood function, $\mathcal{L}$; this is computationally infeasible to produce exactly. We thus settle for a multivariate Gaussian approximation, and estimate the relative log-likelihoods per degree of freedom in this limit. This gives an estimate of how more strongly preferred the base model is to its variants.

Given the predictions of a particular model we would like to quantify how well they reproduce the data. Doing so requires us to estimate how the observables, which we write schematically as $\{x_i\}$, would change, and how those changes would be correlated with each other, were another set of observations made of the same group under sufficiently similar conditions.

Different simulations imply different noise models, with different correlations. We estimate the intrinsic variation of the data from the simulations themselves, taking many sequences of length $N = 1096$ fights, and estimating the statistics – the various $P(A)$, $P_c(AB)$, $\bar{n}(A)$, $\bar{n}(AB)$ and ΔP – from them in the same fashion as in the actual data. We thus reduce the problem to estimating the shape of the likelihood function – the probability that a measurement drawn from a particular model H will take the values $\{x_i\}$, which we write as $L(\{x_i\}|H)$. When there are only a few x_i to measure, the function can be sampled. Yet for the statistics of interest here, L is a function with potentially thousands of dimensions (for example, the 1128 pairs, in the case of $P_c(AB)$ and $\bar{n}(AB)$, or the 2304 tuples, in the case of $\Delta P(A \to B)$.)

The standard solution to this problem is to expand the logarithm of the likelihood to second order about the maximum; this amounts to approximating the distribution of the n statistics as a multivariate Gaussian. For a simultaneous measurement of n statistics – say, for example, the 1128 pair probabilities of a $P_c(AB)$ measurement – the second order expansion is

$$\begin{aligned}\mathcal{L} &= -\ln L(\{x_i\}|H) \\ &= \frac{n}{2}\ln 2\pi - \frac{1}{2}\det\Sigma + \frac{1}{2}\Delta_i\Sigma_{ij}\Delta_j + \ldots,\end{aligned} \quad (5)$$

where repeated indices indicate summation, Δ_i is

$$\Delta_i = (x_i - \bar{x}_i), \quad (6)$$

and $\bar{x}$, the value the measurements that maximizes L, and Σ, sometimes called the precision matrix, are a set of parameters we estimate from the simulations. The first two terms, the zeroth order part of the expansion, are what ensure that the likelihood L is normalized properly so that $\int L(\{x_i\})(\prod dx_i)$ is unity.

We can estimate $\bar{x}$ and Σ by the average values, and the inverse of their covariance matrix, of a set of A simulation runs. It is required that $A > n$ for the covariance matrix to be invertible. We can then compare two models – say, *Base* and *Variant* – by taking likelihood ratios – which is more convenient to do in log space:

$$\Delta\mathcal{L} = \mathcal{L}(\{x_i^D\}|\text{Base}) - \mathcal{L}_{H1}(\{x_i^D\}|\text{Variant}), \tag{7}$$

where $\{x_i^D\}$ are measured from the data. The larger (more positive) $\Delta\mathcal{L}$, above, is, the greater the likelihood of the variant; conversely, negative values of $\Delta\mathcal{L}$ are associated with a higher likelihood attributed to the base model.

Table 2. $\Delta\mathcal{L}/n$, log-likelihood per observable, relative to the *Base* model, for the model variants.

Model $\Delta\mathcal{L}/n$	$P(A)$	$P_c(AB)$	$\bar{n}(A)$	$\bar{n}(AB)$	ΔP $A \to B$	Overall
Shuffled						
Total	-6.0	-15	-8.2	-5.5	+0.58	**-6.7**
Outgoing	-7.9	+0.78	+3.2	-0.06	-25	**-6.7**
Incoming	-0.61	+0.58	-13.3	-8.0	+0.37	**-3.5**
Coarse Grained						
$n = 1$	-7.7	-7.3	-9.7	-2.2	-3.1	**-5.3**
$n = 2$	-2.6	-9.7	-2.3	-1.6	-2.2	**-4.0**
$n = 4$	-3.3	-7.6	+2.4	-0.12	+0.08	**-2.0**

For observables of pair properties such as $P_c(AB)$, only the top 100 pairs are considered. In general, individual log-likelihoods are negative, indicating that the base model is preferred over the variants for different subsets of the data; the overall log-likelihoods are all negative.

Table 2 shows the results, for all 48 individuals (in the case of $P(A)$ and $\bar{n}(A)$), the top 100 tuples (in the case of $P_c(AB)$ and $\bar{n}(AB)$), or the top 100 "ordered pairs with repetitions allowed" (in the case of $\Delta P(A \to B)$.) In general, the entries are negative, indicating that the base model has higher likelihood than the shuffled models. In some cases, $\Delta\mathcal{L}/n$ is positive; indicating that one of the variants has a slightly higher-likelihood for a particular class of measurements. There is, however, no variant that consistently outperforms the base model, and the overall log-likelihoods (computed in the approximation that the different classes of measurements are independent) are all negative.

Model Complexity

Some of the models that fit the data poorly – in particular, the two variants of $\mathcal{C}(1,1)$ – have far fewer parameters than our favored model, $\mathcal{C}(2,1)$+`AND`. There are, formally, 54,144 free parameters in the latter model, while the simpler models have only 2,304. In this section, we investigate, in two different ways, whether the improvements in goodness-of-fit, though large, are justified in an information-theoretic sense.

Our first intuition is that many of the parameters in $\mathcal{C}(2,1)$+`AND` have little influence on the evolution of the system; for example, setting some of the smaller ΔP values to zero has little effect on the evolution of the system (see the Supporting Information model specification.) Here, we use the notion of Bayesian complexity to estimate the true number of free parameters; we then use an information theoretic criterion to justify our favorable assessment of $\mathcal{C}(2,1)$.

Bayesian complexity (see Ref. [1] and, *e.g.*, Ref. [2,3]) provides one way to measure the "effective"

number of free parameters, $k_{\rm eff}$. It can be written

$$k_{\rm eff} = 2(\ln L(\hat{\theta}) - \overline{\ln L(\theta)}), \tag{8}$$

where the overbar denotes average with respect to the posterior PDF and $\hat{\theta}$ are the best estimates of the parameters of the model, θ.

Without a fuller exploration of parameter space, we can not estimate $\overline{\ln L}$ to great accuracy. However, if we assume that many of our parameters will be degenerate – *i.e.*, that our likelihood is reasonably flat – then the average likelihood can be approximated by the likelihood at a single "average" position within the *prior* distribution.

If we take as our prior only the distribution of ΔP values, then an average point in strategy space is given by the *Total* shuffle, described in Results section of the main paper. We then compute the log-likelihood for the 54,144 values of $N(AB \rightarrow C)$ (directly related to the ΔP values of the model, these are quicker to compute.) Computing the multivariate Gaussian approximation to the likelihood function for such a large set of parameters is computationally infeasible (it would require over $50,000$ simulation runs), and so we approximate the likelihood as diagonal and Poissonian; because many of the observed frequencies are small (of order one detection in a set of 100 simulation runs of 1000 fights each), we use the functional form of the true Poisson distribution and not standard the Gaussian approximation. We can check to see that the measured covariances are Poissonian (*i.e.*, roughly equal to the measured mean); they are, within the error expected for a measured covariance.

We find with this method that $k_{\rm eff}$ is ~ 2000. Compared to the formal number of 54,144, this back-of-the-envelope calculation suggests that our intuition – that many of the parameters of the model are underdetermined and are thus not truly observable – is correct.

In general, adding more parameters will increase the goodness of fit – regardless of whether an underlying model requires such complexity; one may, for example, be fitting noise and not signal. There number of information-theoretic statistical tools to penalize model complexity. The *Akaike Information Criterion* (AIC; [4]), based on the relative Kullback-Leibler information, is one of the best known. Discussed in detail in (for example) Ref. [3, 5], the AIC value is

$$\mathrm{AIC} = -2\ln L + 2K + \frac{2K(K+1)}{N-K-1} \tag{9}$$

where K is the number of parameters in the model and N is the number of observables. A lower AIC value is better, and indicates the preferred model (only relative AIC values are meaningful.)

We can now compare $\mathcal{C}(2,1)$ with $\mathcal{C}(1,1)$; in particular, we can find an lower bound on the AIC for the $\mathcal{C}(1,1)$ model by assuming its number of effective parameters to be much less than the 2000 found for $\mathcal{C}(2,1)$.

We find that $\ln L/N$ is -1.1 for $\mathcal{C}(2,1)$+`AND`, and -1.9 for $\mathcal{C}(1,1)$+`OR`; the overall difference in log-likelihood between the two models is then $\sim 80,000$. The complexity penalty is $2K + 2K(K+1)/(N-K-1) \sim 4000$. This means that, despite the complexity of added parameters, $\mathcal{C}(2,1)$+`AND` is significantly preferred over the simpler $\mathcal{C}(1,1)$+`OR`.

References

1. Spiegelhalter D, Best N, Carlin B, Linde A (2002) Bayesian measures of model complexity and fit. J Roy Stat Soc B 64: 583–639.

2. Kunz M, Trotta R, Parkinson DR (2006) Measuring the effective complexity of cosmological models. Phys Rev D 74: 23503.

3. Liddle AR (2007) Information criteria for astrophysical model selection. Mon Not R Astron Soc Lett 377: L74.

4. Akaike H (1978) A Bayesian analysis of the minimum AIC procedure. Ann I Stat Math .

5. Burnham K, Anderson D (2004) Multimodel inference: Understanding AIC and BIC in model selection. Sociol Method Res 33: 261.

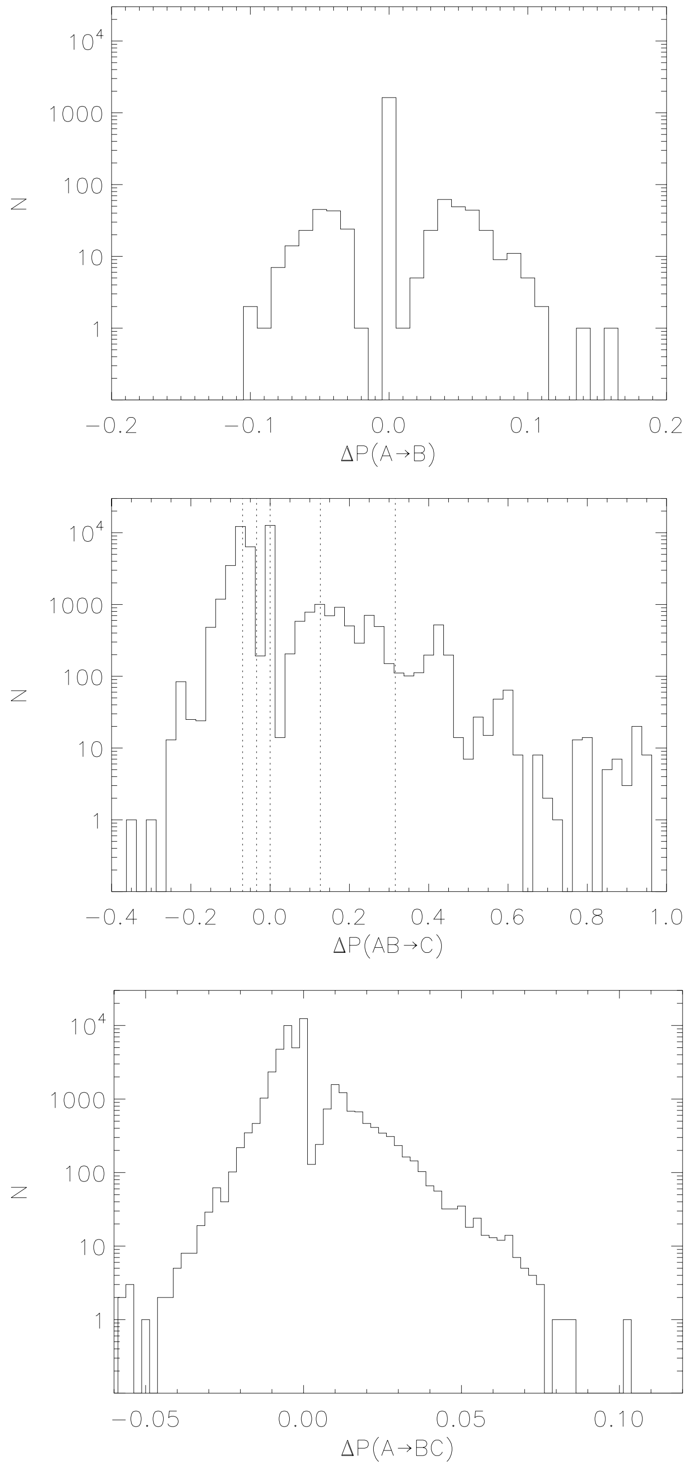

Figure 4. The distribution of correlations $\Delta P(A \rightarrow B)$, $\Delta P(AB \rightarrow C)$, and $\Delta P(A \rightarrow BC)$ in the observed data. These quantify the probability that an appearance of a particular individual or group will be associated with a later appearance (or absence) of another individual or group. The dotted lines, overlaid on the $\Delta P(AB \rightarrow C)$ distribution, are referred to in the strategy specificity subsection of the Results; they indicate the values to which probabilities are coarse-grained at two-level resolution. $\Delta P(AB \rightarrow C)$ values are larger since they are normalized by $N(AB)$ and not $N(A)$.

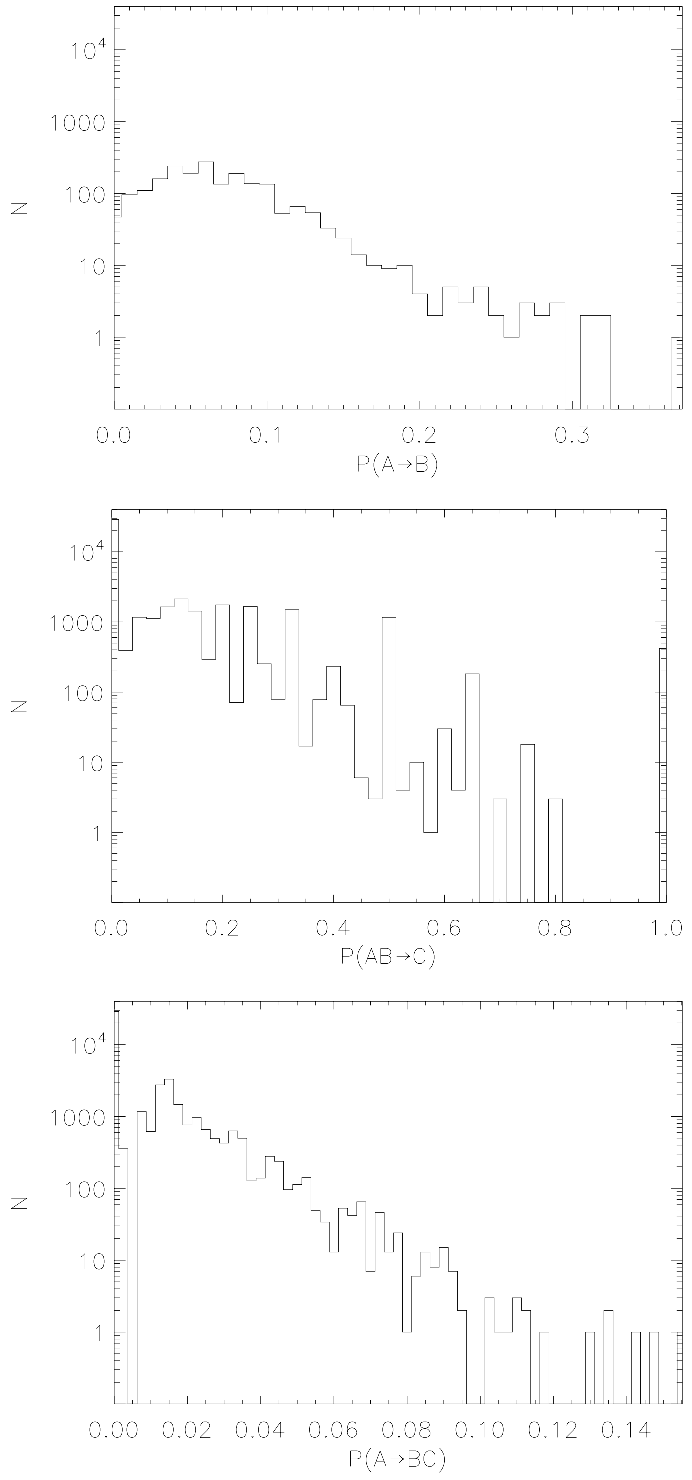

Figure 5. As in Fig. 4, but now showing the distribution of the absolute probabilities, $P(A \to B)$, $P(AB \to C)$ and $P(A \to BC)$.

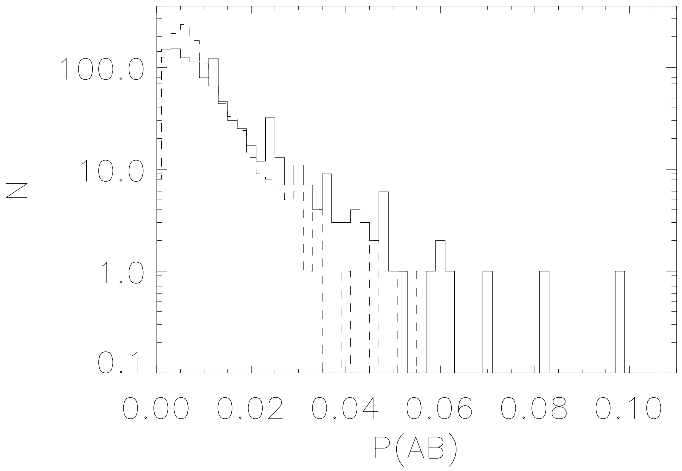

Figure 6. The distribution of measured joint appearance probability, $P(AB)$ [solid line], and that expected in the case that individuals appear in fights independently of each other [dotted line]. The two distributions are significantly different under the Kolmogorov-Smirnov test.
